# TET1 inhibits cell proliferation by inducing RASSF5 expression

**DOI:** 10.18632/oncotarget.21189

**Published:** 2017-09-23

**Authors:** Bo-Tai Li, Chao Yu, Ying Xu, Sheng-Bing Liu, Heng-Yu Fan, Wei-Wei Pan

**Affiliations:** ^1^ Department of Cell Biology, College of Medicine, Jiaxing University, Jiaxing 314001, China; ^2^ Life Sciences Institute, Zhejiang University, Hangzhou 301158, China

**Keywords:** ovarian cancer, TET1, RASSF5, proliferation, DNA methylation

## Abstract

Tet methylcytosine dioxygenases (TETs) catalyze the oxidative reactions of 5-methylcytosine to 5-hydroxymethylcytosine (5hmC). However, TET1 roles in ovarian cancer cell growth are unknown. Here, we show that ectopic expression of TET1 increased 5hmC levels, and inhibited proliferation and colony formation in ovarian cancer cell lines. Furthermore, *in vitro* and *in vivo* functional studies demonstrated that TET1 overexpression is necessary for the suppression of ovarian cancer growth, whereas depletion of *TET1* expression had the opposite effect. Furthermore, the results of RNA-seq and qRT-PCR analyses identified a tumor suppressor, Ras association domain family member 5 (*RASSF5*), as the key downstream target of TET1. TET1 promotes RASSF5 expression by demethylating a CpG site within *RASSF5* promoter. Up-regulated RASSF5 expression leads to the suppression of ovarian cancer cells growth. Additionally, we demonstrated that inhibition of CUL4-DDB1 ubiquitin ligase complex decrease 5hmC levels in ovarian cancer cells. These results provide new insights into the understanding of how ovarian cancers develop and grow, and identify TET1 as a key player in this process.

## INTRODUCTION

Ovarian cancer is the leading cause of death from all gynecological cancers [1]. However, mechanisms and factors regulating ovarian cancer growth and metastasis remain to be completely elucidated. Epigenetic alterations have long been linked to ovarian cancer initiation and progression, and they include DNA methylation changes and histone modifications. The best-characterized epigenetic mechanism is DNA methylation [2], which is crucial for gene silencing and transcriptional regulation of repetitive elements. DNA methylation occurs predominantly at CpG dinucleotides where DNA methyltransferases (DNMTs) mediate the transfer of a methyl group to cytosines, generating 5-methylcytosine (5mC) [3, 4]. Abnormal DNA methylation can lead to the development of genetic diseases such as Prader-Willi syndrome [5]. Moreover, during carcinogenesis, the methylation of CpG sites in the promoter region, together with genome-wide hypomethylation, leads to the silencing of tumor suppressor genes and activation of oncogenes, promoting cancer initiation and progression [6–8].

Previously, 5mC was considered the only modified base in animals, until the discovery of 5-hydroxymethylcytosine (5hmC) in bacteriophage and mammal DNA [9]. This epigenetic modification is catalyzed by the tet methylcytosine dioxygenase (TET) enzymes [10]. TET proteins mediate the sequential oxidation of 5mC to 5hmC, leading to DNA demethylation [11]. Using anti-5hmC antibody-based immuno-dot-blot, it was demonstrated that 5hmC levels are greatly reduced in multiple cancer types, such as squamous cell lung cancers and brain tumors [12, 13], but the functional significance of this process has not been elucidated.

The TET family of DNA hydroxylases, which includes TET1, TET2, and TET3, mediates the conversion of 5mC to 5hmC. TET expression was shown to be dysregulated in multiple malignances, including breast cancer, hepatocellular carcinoma, melanoma, and glioma [14–16]. *TET1* was firstly identified as a fusion partner of mixed lineage leukemia (MLL) in acute myeloid leukemia (AML) [17, 18], and the downregulation of *Tet1* has been shown to promote cancer invasion and metastasis [19, 20]. *TET2* gene was found to be mutated in cancers, leading to the loss of 5hmC [21]. Even though TET proteins have been shown to have an important role in cancer development, their roles in ovarian cancer are largely unknown.

In this study, we demonstrated that *TET1* expression is downregulated in most ovarian cancer tissues and cells. Therefore, we examined how the dysregulation of *TET1* expression affects carcinogenesis, and aimed to determine the molecular mechanisms underlying these processes.

## RESULTS

### 5hmC levels are decreased in ovarian cancer tissue and cells

**Figure 1:**
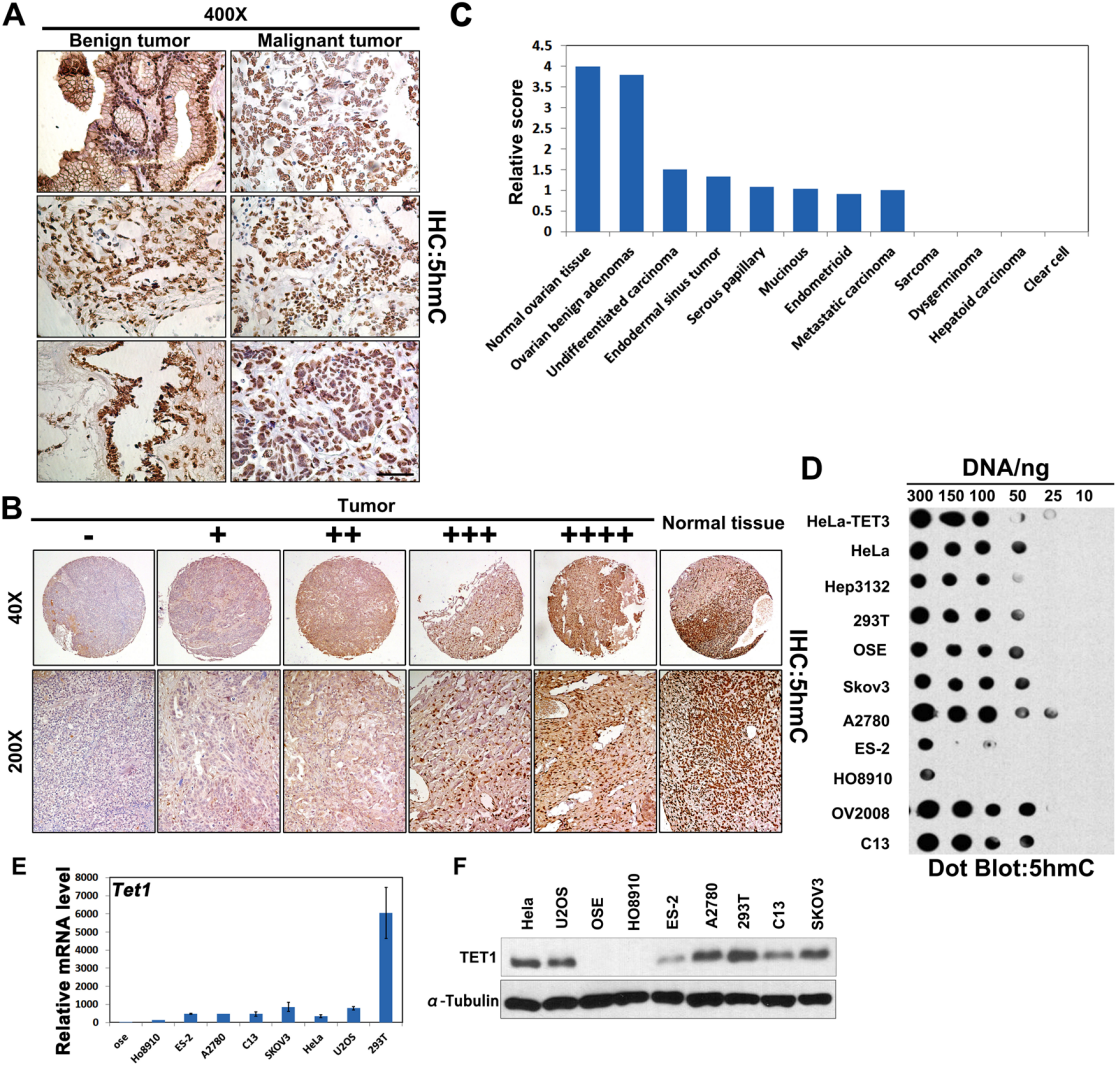
5hmC production in human ovarian cancer samples and cells **(A)** IHC analysis of 5hmC levels in human ovarian benign (n=5) and malignant tumor tissue samples (n=10). Sections were counterstained with hematoxylin. Scale bar, 50 μm. **(B)** Representative image of 5hmC staining (brown) in human ovarian cancer tissue samples with normal ovarian tissue (n=177). **(C)** Quantification of relative TMA scores of the described human samples. **(D)** 5hmC expression in ovarian cancer cells (SKOV3, A2780, ES-2, HO8910, OV2008, and C13), Hep3132, 293T, immortalized mOSE cells, and HeLa cells *in vitro*. HeLa-TET3 was used as positive control. **(E)** Quantitative RT-PCR analysis of *TET1* expression in the indicated cancer cells or primary cells. Results are represented as mean ± standard error (SE) obtained in three independent experiments. **(F)** SKOV3, A2780, ES-2, HO8910, C13, U2OS, 293T, immortalized mOSE cells, and HeLa cells were subjected to immunoblot (IB) analysis with antibodies to TET1 and α-Tubulin proteins.

The levels and distribution of 5hmC in ovarian benign and malignant tumors were determined. As presented in Figure 1A, 5hmC levels were shown to be significantly higher in benign, compared with those in the malignant tumor samples. To confirm these results, we used two tissue arrays containing four normal ovarian tissue samples, five benign adenomas, 74 serous cystadenomas, 31 mucinous cystadenomas, 53 endometrioid carcinomas, and one clear cell cystadenoma sample (Table 1 and Supplementary Tables 1 and 2). Based on staining intensity, we classified the samples into five groups with increasing staining intensity from the weakest (-) to the strongest (++++; Figure 1B). As summarized in Figure 1C, strong nuclear 5hmC signal was observed in normal human ovarian samples and benign adenomas, whereas partial or complete loss of 5hmC staining was seen in almost in all primary and metastatic ovarian cancer cells.

**Table 1:** 5hmC protein expression in ovarian cancer and normal ovary tissues

	Grade					
Ovarian cancer type	Case(*N*)	++++	+++	++	+	-
Normal ovarian tissue	4	4(100%)				
Ovarian benign adenomas	5	4(80%)	1(20%)			
Undifferentiated carcinoma	2			1(50%)	1(50%)	
Endodermal sinus tumor	3			1(33.33%)	2(66.67%)	
Serous papillary	74	5(6.76%)	4(5.41%)	10(13.5%)	28(37.84%)	27(36.49%)
Mucious	31		6(19.35%)	4(12.91%)	6(19.36%)	15(48.39)
Endometrioid	53		4(7.55%)	10(18.86%)	16(30.19%)	23(43.4%)
Hepatoid carcinoma	1				1(100%)	
Metastatic carcinoma	1					1(100%)
Sarcoma	1					1(100%)
Dysgerminoma	1					1(100%)
Clear cell	1					1(100%)

Furthermore, we purified genomic DNA from ovarian cancer cells (SKOV3, A2780, ES-2, HO8910, OV2008, C13), other types of cancers (HeLa, Hep3132), or mOSE cells, and detected 5hmC levels using anti-5hmC antibody-based dot-blotting. The levels of this molecule were shown to be low in ES-2 and H08910 cells, but high in A2780 and OV2008 cells. HeLa cells transfected with TET3 plasmids were used as the positive control (Figure 1D), and these results demonstrated that 5hmC levels differ between ovarian cancer cell types. We confirmed that *TET1* mRNA level was weakly expressed in HO8910 and ES-2 cells by quantitative RT-PCR (Figure 1E). At the protein level, as compared with other cells, TET1 was highly expressed in A2780 cells, but it was weakly expressed in ES-2 cell (Figure 1F), which was consistent with the observed 5hmC levels in ovarian cancer cells.

### Knockdown of TET1 or CUL4-DDB1 ubiquitin ligase complex decrease 5hmC levels in ovarian cancer cells

**Figure 2:**
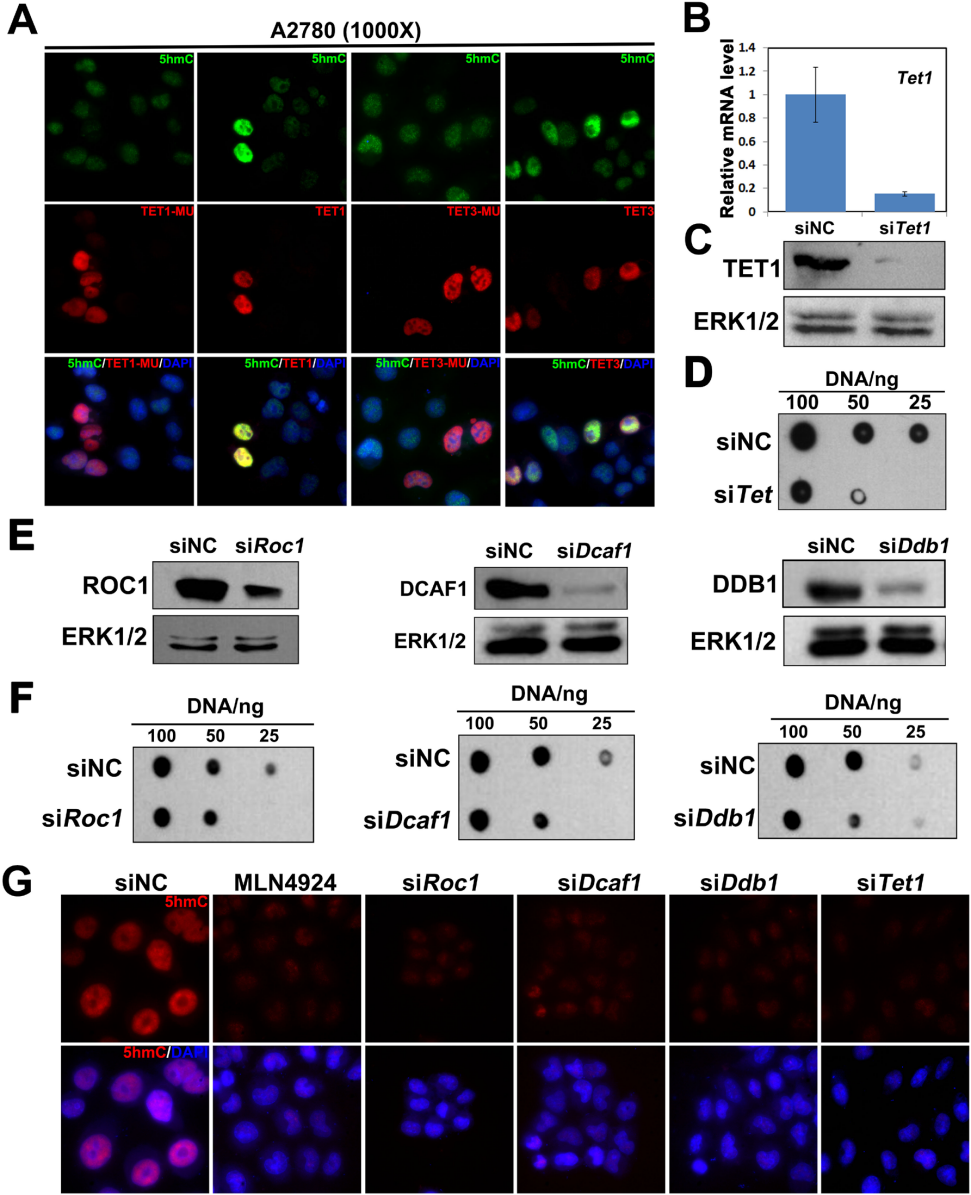
TET1 and CUL4-DDB1 ubiquitin E3 ligase complex depletion decrease 5hmC levels in ovarian cancer cells **(A)** A2780 cells transfected with TET1, TET3, TET1 mutant (TET1-MU) and TET3 mutant (TET3-MU) plasmid were subjected to immunofluorescenceanalysis with antibodies to the indicated protein. **Green**, 5hmC; **Red**, Flag-labeled TET1 and TET3-overexpressing cells or TET1-MU and TET3-MU overexpressing cells; **Blue**, DAPI-stained DNA. **(B)** A2780 cells transfected with either siRNA negative control (siNC), or si*TET1* were subjected to quantitative RT-PCR analysis of *TET1* mRNA level. **(C)** A2780 cells transfected with either siRNA negative control (siNC), or si*TET1* were subjected to immunoblot analysis with antibodies to TET1 proteins. **(D)** A2780 cells transfected with either siNC, or si*TET1* were subjected to Dot-blot analysis of 5hmC levels. **(E)** A2780 cells transfected with siNC, si*ROC1, siDCAF1*, or si*DDB1* were subjected to immunoblot analysis with antibodies to the indicated protein. **(F)** A2780 cells transfected with siNC, si*ROC1, siDCAF1*, or si*DDB1* were subjected to Dot-blot analysis with antibodies to the indicated protein. **(G)** Immunofluorescence results, showing that *ROC1*, *DCAF1*, *DDB1*, and *TET1* knockdown, and treatment with MLN4924 lead to the reduction of 5hmC levels in A2780 cells. **Red**, 5hmC; **Blue**, DAPI-stained DNA. A2780 cells transfected with siNC, si*ROC1*, si*DCAF1*, si*DDB1* or si*TET1* and treatment with MLN4924 were subjected to immunofluorescence analysis with antibodies to the indicated protein.

TET1 is responsible for 5mC to 5hmC conversion, and we attempted to elucidate the effects of *TET1* overexpression on 5hmC levels in ovarian cancer cells. We overexpressed *TET1* or *TET3* in A2780 cells and determined the levels of 5hmC using immunofluorescence staining. We observed a global increase in 5hmC levels in *TET1* or *TET3*-overexpressing cells, but not in TET1-MU- or TET3-MU-overexpressing cells (Figure 2A). To demonstrate that TET increase 5hmC levels further, the effects of TET1 depletion in ovarian cancer cells were analyzed. Compared with control, si*TET1* cells expressed significantly lower levels of TET1 (Figure 2B and 2C). Moreover, knockdown of TET1 significantly decreased 5hmC levels in A2780 cells (Figure 2D).

To identify the roles of CUL4-DDB1 E3 ligase complex in ovarian cancer cells, we performed a knockdown of CUL4-DDB1 E3 ligase components, ring of cullin-1 (*ROC1)*, *DDB1*, or DDB1/CUL4-associated factor-1 (*DCAF1*) in A2780 cells, and detected 5hmC levels by dot-blot assays (Figure 2E and 2F). We found that the knockdown of these genes leads to a reduction in 5hmC levels in ovarian cancer cells. Moreover, MLN4924 was shown to be a potent inhibitor of cullin neddylation, and to inactivate CUL4-DDB1 E3 ligase complex, leading to the accumulation of its substrates [22, 23]. Here, MLN4924 treatment led to a decrease in 5hmC levels as well (Figure 2G).

### TET1 inhibits ovarian cancer cell growth and colony formation *in vitro*

**Figure 3:**
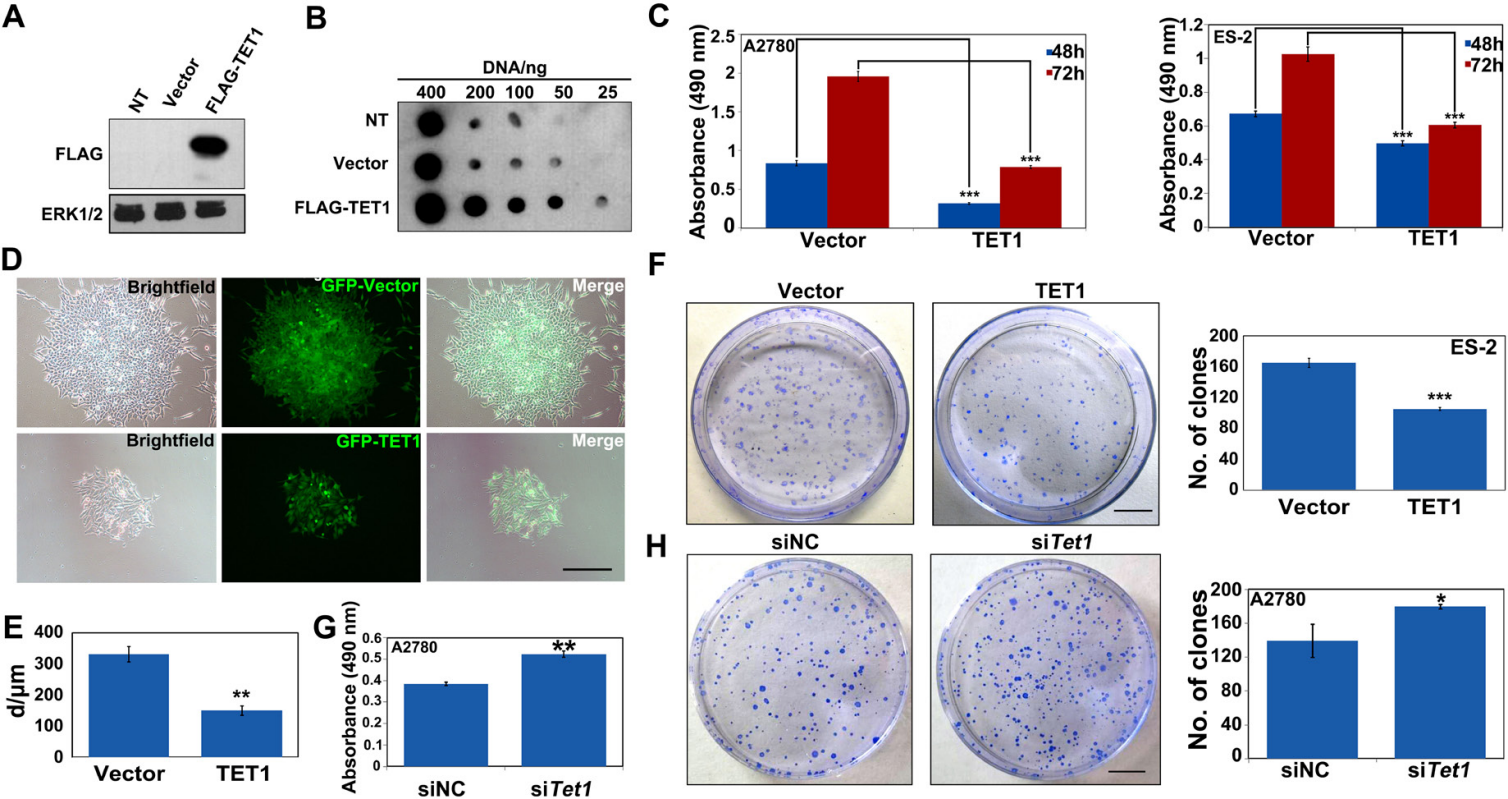
TET1 overexpression inhibits ovarian cancer cell proliferation and colony formation *in vitro* **(A)** *TET1*-overexpressing ES-2 cells and control cells were subjected to immunoblot analysis with antibodies to the indicated protein. No treatment cell (NT). **(B)** *TET1*-overexpressing ES-2 cells and control cells were subjected to Dot-blot analysis of 5hmC levels. **(C)** *In vitro* proliferation of *TET1*-overexpressing A2780 and ES-2 cells. 5×10^3^ cells were plated in 96-well culture dishes in triplicate and followed by MTT assay. Data are means ±SE of triplicate cultures from a representative experiment. **(D)** Abundant GFP signals were observed using fluorescence microscope. Left panel: bright field image. Middle panel: dark field image. Right panel: merge. Scale bar, 50 μm. **(E)** Quantitative analysis of the results presented in D. **(F)** Colony formation of ES-2 cells, with or without *TET1* overexpression. Colony numbers and colony diameter were counted at day 10. Scale bar, 50 μm. Data are means ±SE of triplicate cultures from a representative experiment. **(G)** *In vitro* proliferation of *TET1*-knockdown A2780 cells. siNC and si*TET1* cells were plated in 96-well culture dishes in triplicate and followed by MTT assay. Data are means ±SE of triplicate cultures from a representative experiment. **(H)** Colony forming of si*TET1*-A2780 cells. Results are represented as mean values ± SEs obtained in three independent experiments. Scale bar, 50 μm. *** *p*<0.001; ** *p*<0.01; * *p*<0.05.

To elucidate the role of TET1 in ovarian cancer cell growth, ovarian cancer cells were infected with lentiviral LV-CON or LV-TET1. The expression of GFP-TET1-Flag was confirmed by western blot analysis (Figure 3A). The levels of 5hmC were shown to be significantly increased in TET1-overexpressing ovarian cancer cells, which was consistent with the results obtained using transient TET1 or TET3 overexpression (Figure 3B and 2A). We next analyzed the effect of TET1 overexpression on ovarian cancer cell proliferation and showed that this leads to a remarkable reduction in cell growth, compared with that in the control samples (Figure 3C). TET1-overexpressing ES-2 cells were shown to form a significantly lower number of colonies and their colony sizes were smaller than those in the control cells (Figure 3D-3F). In addition, *TET1 gene* silencing promoted A2780 cells growth and increase colonies number by colony forming assay (Figure 3G and 3H).

### TET1 suppresses tumor growth and promotes cancer cell apoptosis *in vivo*

**Figure 4:**
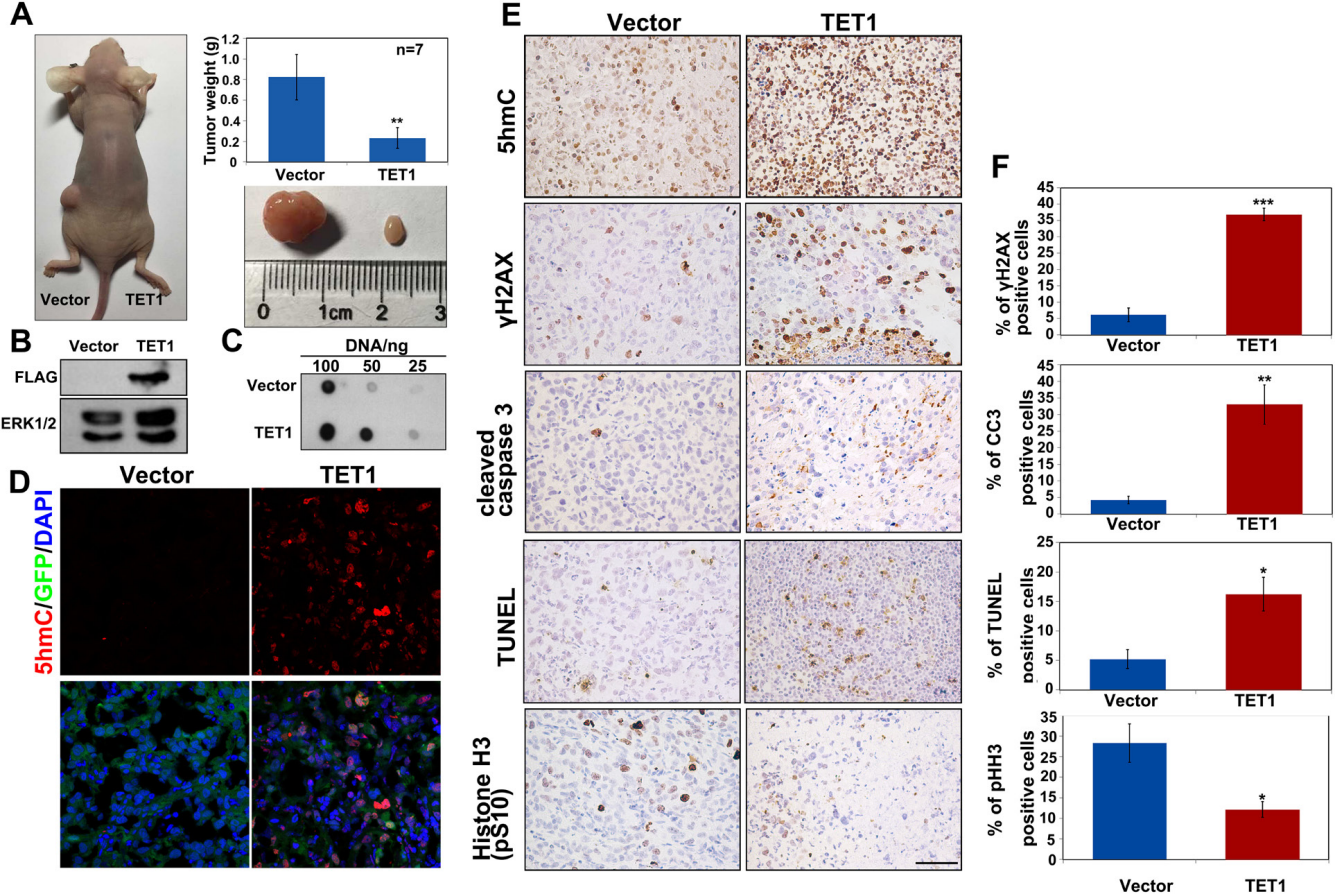
TET1 overexpression in tumors inhibits tumor growth *in vivo* **(A)** Equal numbers of ES-2 infected with LV-CON or LV-GFP-TET1-Flag were implanted subcutaneously into the left and right flanks of nude mice, respectively. Tumors were removed and weighed after 30 days. n=7 tumors for each group. Results are represented as mean values ± SEs obtained in seven independent experiments. **(B)** Flag-tagged protein expression in tumor tissues that developed from the implanted ES-2 cells. **(C)** The levels of 5hmC in tumor tissues that developed from the implanted ES-2 cells. **(D)** Levels of 5hmC and TET1 in cryosections prepared from ES-2 cell-derived tumor tissues. **Red**, 5hmC. **Green**, TET1. **Blue**, DAPI staining. **(E)** IHC analysis of 5hmC, γH2AX, cleaved caspase 3, and phospho-histone H3 levels, and TUNEL staining of ES-2-derived tumor tissue samples. Scale bar, 50 μm. **(F)** Quantitative of percentage of the positive cell in (E) was performed in three randomly chosen fields for each group.

To evaluate the role of TET1 in tumor growth *in vivo*, we subcutaneously transplanted equal numbers of control ES-2 cells or ES-2 cells stably expressing TET1 into the left and right flanks of nude mice, respectively. We found that TET1-overexpressing tumors were significantly smaller than those in the control group. The weight of the tumors formed by TET1-overexpressing cells was four-fold lower than that of the control cells (Figure 4A). To confirm the transplanted cells are responsible for the tumor growth, western blot analysis demonstrated that most of the *TET1*-overexpressing tumor cells were positive for Flag-TET1 (Figure 4B). Afterwards, we determined 5hmC levels in tumor tissue samples and demonstrated that they were dramatically increased in *TET1*-overexpressing tumors (Figure 4C and 4D). Previous studies showed that TET1 can activate PARP-1/ARTD1 independently of DNA breaks and induce tumor cells apoptosis in multiple tissue types [24]. To elucidate the effects of TET1 on the ovarian cancer cell apoptosis, we measured pH2AX and cleaved caspase 3 levels by IHC and immunofluorescence staining. We found that both pH2AX and cleaved caspase 3 were highly expressed in *TET1*-overexpressing tumor. The levels of cell proliferation marker, phospho-histone H3, were shown to be decreased in *TET1*-overexpressing tumors (Figure 4E and 4F, and Supplementary Figure 1A-1C). Additionally, no significant difference in the expression of collagen IV between the analyzed tumor tissue samples was observed, indicating that TET1 may not be involved in tumor angiogenesis (Supplementary Figure 1D).

**Figure 5:**
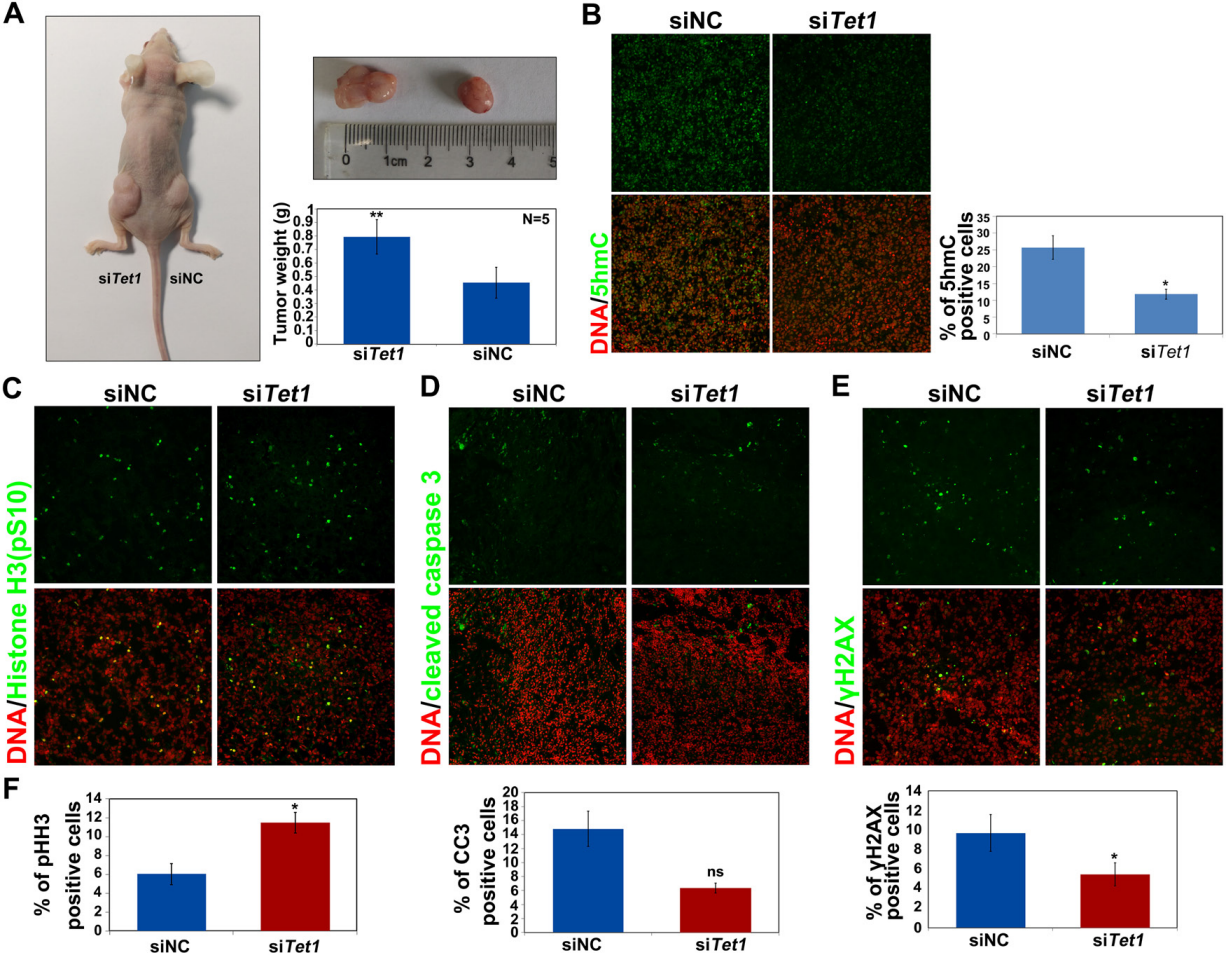
*TET1* gene silencing in tumors promotes tumor growth *in vivo* **(A)** A2780 cells, transfected with either siNC or si*TET1*, were injected subcutaneously into the right and left flanks of nude mice, respectively, and the resulting tumor tissue was removed and weighed (n=5). Results are represented as mean values ± SEs obtained in five independent experiments. **(B)** Immunofluorescence analysis of 5hmC levels (**green**) in tumor tissues with or without *TET1* expression. Quantitative of percentage of the positive cell in B was performed in three randomly chosen fields for each group. **(C-E)** Immunofluorescence analysis of phospho-histone H3, cleaved caspase 3 (CC3), and γH2AX levels in tumor tissues, with or without *TET1* expression. **(F)** Quantitative of percentage of the positive cell in (C-E) was performed in three randomly chosen fields for each group. ns, not significant.

To analyze whether the endogenous TET1 expression is important for tumor growth, the expression of *TET1* was inhibited using siRNA in ovarian cancer cells, and the control or *TET1*-knockdown cells were injected into the right and left flanks of nude mice, respectively. The obtained results demonstrated that the inhibition of *TET1* expression in ovarian cancer cells induces tumor proliferation *in vivo* (Figure 5A). si*TET1* tumor weights were shown to be two-fold higher than those of the tumors formed by injecting siNC cells. Furthermore, we confirmed that 5hmC levels were considerably decreased in si*TET1* tumors (Figure 5B). We next examined cleaved caspase 3, pH2AX, and phospho-histone H3 levels, using immunofluorescence analysis, which showed that pH2AX levels were significantly decreased, while the levels of phospho-histone H3 were increased in si*TET1* tumors, in comparison with those in the control samples. No significant difference in cleaved caspase 3 levels was observed between si*TET1* and siNC tumors (Figure 5C-5F), and collagen IV levels did not change between si*TET1* and siNC tumors (Supplementary Figure 1E).

### TET1-dependent demethylation promotes transcriptional activation of *RASSF5*

**Figure 6:**
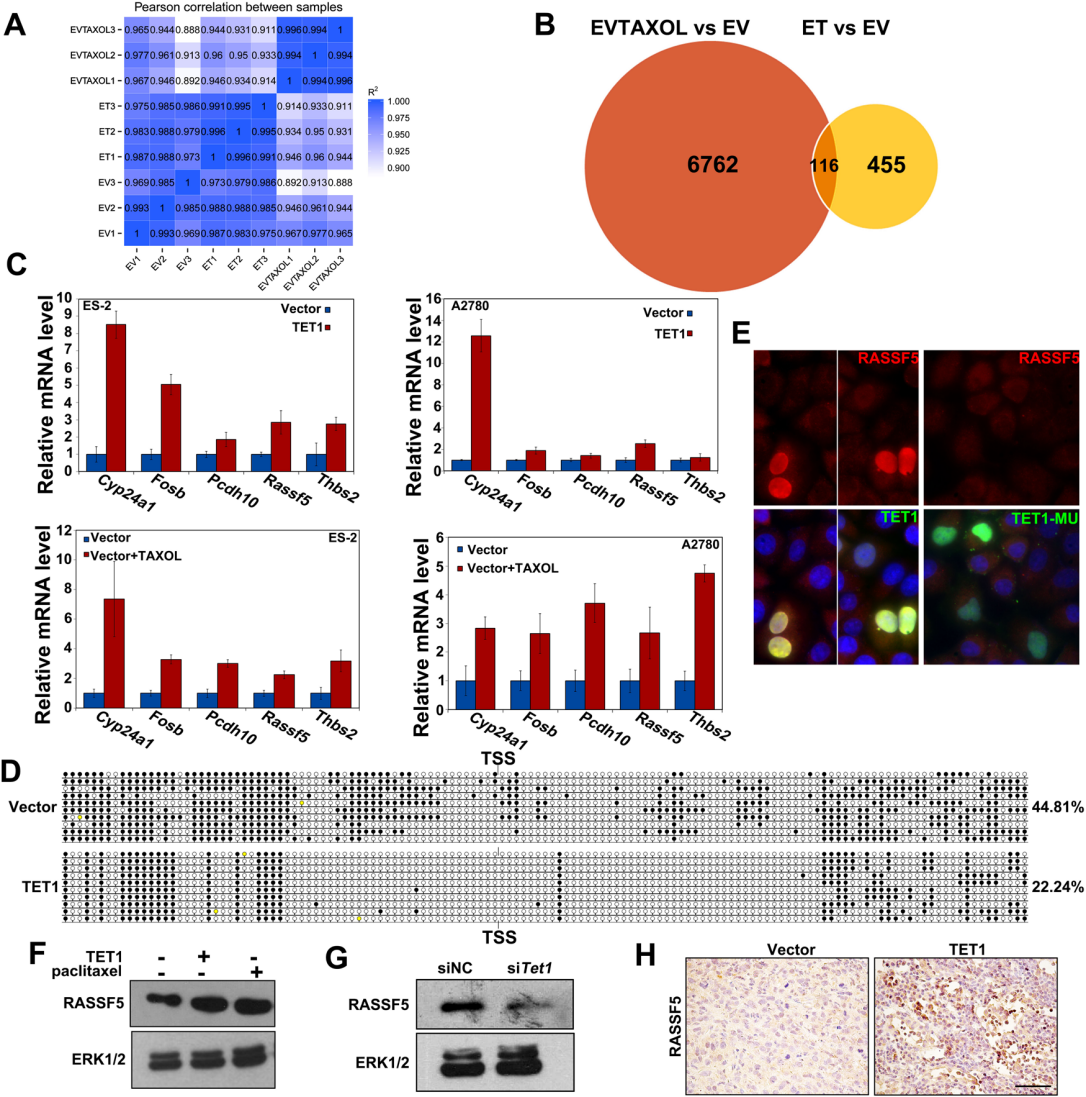
*RASSF5* is upregulated in *TET1*-overexperessing ovarian cancer cells and tumors **(A)** Genome-wide RNA-seq analysis of gene expression levels in EVTAXOL, ET, and EV cells. **(B)** Upregulated genes in EVTAXOL and ET groups, in comparison with those in the EV group. **(C)** qRT-PCR confirm up-regulated gene in EVTAXOL and ET groups. **(D)** TET1 overexpression decrease *RASSF5* promoter methylation in ovarian cancer cells. Open and filled circles represent unmethylated and methylated CpG islands, respectively. The percentages of methylated CpGs are indicated. **(E)** Immunofluorescence analysis of RASSF5 (**red**) levels in ovarian cancer cells. **Green**, Flag-labeled TET1 and TET1-MU overexpressing cells. **Blue**, DAPI staining. **(F, G)** Western blot results, showing RASSF5 levels in *TET1*-overexpressing, paclitaxel-treated, and si*TET1*-treated cells. **(H)** IHC staining, showing RASSF5 levels in *TET1*-overexpressing tumor tissue samples.

To understand how TET1 expression inhibits ovarian cancer growth, we performed a genome-wide RNA-seq analysis of total mRNA isolated from ES-2 cells treated with paclitaxel (0.01 μg/mL) (EVTAXOL) or without it (EV) for 24 h and from *TET1*-overexpressing ES-2 (ET) or empty vector-carrying (EV) cells (Figure 6A). We determined that the expression of 6762 genes was upregulated in EVTAXOL group, 455 genes were upregulated in the ET group, while 116 of these genes were upregulated in both groups (Figure 6B). Further analysis showed that the upregulated genes are involved in cell adhesion or cancer development, such as NF-κB and TNF signaling pathways (Supplementary Figure 1F). In order to confirm these results, we verified the differential expression of several selected genes in ES-2 cells. *CYP24*, *FOSB*, *PCDH10*, *THBS2*, and *RASSF5* were selected as representative genes for further analysis, as they are well-known regulators of cancer cell proliferation and apoptosis, and the results obtained by qPCR analysis were shown to support our previously obtained results (Figure 6C).

RASSF is a family of tumor suppressors, which are frequently inactivated through promoter hypermethylation in various cancers [25–27]. To determine whether TET1 affects CpG methylation of *RASSF5* promoter, we performed conventional bisulfite sequencing and showed that *RASSF5* was ∼44.81% methylated in ES-2 control cells, whereas it was methylated 22.24% in *TET1*-overexpressing ES-2 cells (Figure 6D). No changes in CpG site methylation between the control and *TET1*-overexpressing ES-2 cells were observed when *CYP24A1*, *FOSB*, and *PCDH10* promoters were analyzed (Supplementary Figure 1G). Additionally, to examine whether TET1 overexpression inhibits ovarian cancer growth by increasing RASSF5 levels, the effects of TET1 expression on RASSF5 levels were determined. Using immunofluorescence and western blot analyses, we confirmed that RASSF5 expression is upregulated in *TET1*-overexpressing cells, but not in *TET1*-MU overexpressing cells (Figure 6E and 6F).

Since TET1 overexpression was shown to promote the transcriptional activation of *RASSF5*, we hypothesized that *TET1* silencing using the previously described siRNAs may lead to a decrease in RASSF5 expression. Following this, RASSF5 expression was shown to be downregulated in si*TET1* cells (Figure 6G). Additionally, we observed a significant increase in RASSF5 levels in *TET1*-overexpressing tumor tissue samples (Figure 6H).

### TET1 inhibits ovarian cancer cells growth through an increase in RASSF5 expression

**Figure 7:**
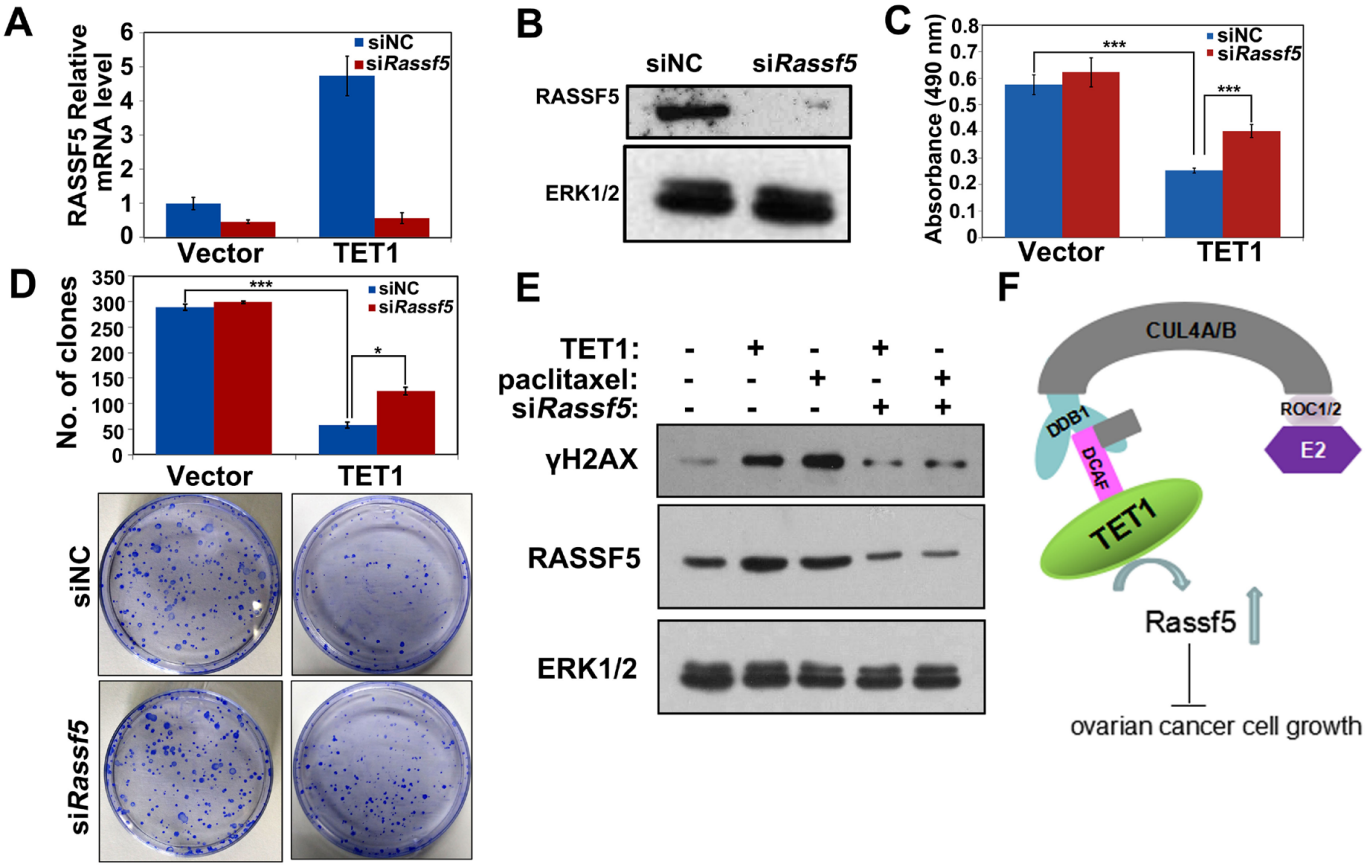
Inhibition of *RASSF5* expression rescues TET1-overexpression-induced phenotype of ovarian cancer cells **(A)** A2780 cells stably expressing control vector and TET1 were transfected with siNT and si*RASSF5*. qPCR detect *RASSF5* mRNA expression. Three replicates are included in this experiment. The error bars represent SEs. **(B)** A2780 cells stably expressing control vector and TET1 were transfected with siNT and si*RASSF5*. Immunoblot analysis was performed with the indicated antibodies. **(C)** Proliferation of *TET1*-overexpressing or control A2780 cells after *RASSF5* silencing. Data are means ±SE of triplicate cultures from a representative experiment. *** *p*<0.001. **(D)** Colony forming assay was performed and the colonies were stained with crystal violet for quantification. Data are means ±SE of triplicate cultures from a representative experiment. *** *p*<0.001; * *p*<0.05. **(E)** RNAi depletion of *RASSF5* rescued the γH2AX levels following *TET1*-overexpression. A2780 cells stably expressing control vector and TET1 were transfected with siNT and si*RASSF5*. Immunoblot analysis was performed with the indicated antibodies. **(F)** Schematic diagram showing TET1 functions in ovarian cancer cells. In ovarian cancer cells, CUL4-DDB1 ubiquitin E3 ligase complex activates TET1, regulate RASSF5 DNA methylation levels and maintain the expressions of *RASSF5* for ovarian cancer cell growth.

To demonstrate that the observed decrease in the tumor cell proliferation rate was due to the TET1-dependent activation of RASSF5, we performed *RASSF5* knockdown experiments. RASSF5 mRNA and protein expression were shown to be efficiently suppressed in A2780 cells and *TET1*-overexpressing A2780 cells (Figure 7A and 7B). *RASSF5* inhibition did not affect A2780 cells growth and colony forming ability by itself, but it was shown to partially rescue TET1-overexpression-induced cell death and colony formation defect (Figure 7C and 7D). In *TET1*-overexpressing cells, *RASSF5* inhibition led to a decrease in pH2AX levels (Figure 7E).

## DISCUSSION

Epithelial ovarian cancer is a heterogeneous disease, comprising many subtypes with distinct clinicopathological and molecular features, and according to these characteristics, they are classified into four major histologic subtypes: serous adenocarcinoma (SAC), mucinous adenocarcinoma (MAC), endometrioid adenocarcinoma (EAC), and clear cell carcinoma (CCC). Although previous studies showed that different histologic subtypes of ovarian cancer have different underlying genetic traits, such as *p53* mutations in SAC, *PTEN* mutations in EAC, and *KRAS* mutations in MAC [28], their epigenetic features have remained elusive. Epigenetic alterations have long been linked to ovarian cancer initiation and progression, including changes in DNA methylation and histone modifications. The best characterized epigenetic marker is DNA methylation/demethylation. In addition to DNMT functions, TET proteins have recently been identified as the novel regulators of DNA methylation. With the increasing number of studies investigating epigenetic alterations and tumorigenesis, TETs and TET-mediated DNA demethylation were shown to play important roles in tumor development and progression [29].

Here, we demonstrated that TET1 effect the methylation of DNA in human ovarian cancer tissues and cells, and that the expression of *TET1* and 5hmC levels were significantly decreased in ovarian cancer tissue and cells, in comparison with those in the normal, healthy tissue. Furthermore, we showed that *TET1* overexpression leads to an increase in 5hmC levels and inhibits cell proliferation and colony formation in several ovarian cancer cell lines, while *TET1* silencing induced the opposite effects *in vitro* and *in vivo*. Our gain- and loss-of-function studies demonstrated that TET1 expression inhibits ovarian tumor growth and promotes apoptosis *in vivo*, while TET1 inhibition in tumor cells promotes tumor growth *in vivo*.

CUL4-DDB1 ubiquitin E3 ligase complexes represent the largest family of E3 ligases and require cullin neddylation for their activation. In our previous study, we determined that CUL4-DDB1 complex regulates mammalian oocyte 5hmC levels by activating TET [30]. We hypothesized that CUL4-DDB1 ubiquitin E3 ligase complexes activates TET, which convert 5mC to 5hmC, and increase DNA demethylation levels in ovarian cancer cells. The results of our studies confirmed that CUL4-DDB1 ubiquitin E3 ligase complexes increase 5hmC production in these cells by acting upstream of TET1, most likely activating it, and determined that the inhibition of CUL4-DDB1 ubiquitin E3 ligase complex expression can lead to a decrease in 5hmC levels. Therefore, results previously obtained by us and other groups indicate that CUL4-DDB1 ubiquitin E3 ligase complexes may affect ovarian cancer cell proliferation and apoptosis, not only by leading to the accumulation of its substrate, DNA replication licensing factor, but through the regulation of epigenetic alterations as well. Neri *et al.* [31] reported that 5hmC levels are considerably reduced in several types of human cancer, such as colon, breast, and prostate cancers. The data obtained in this study demonstrate that 5hmC levels are considerably decreased in ovarian cancer tissue samples and cells. The levels of 5hmc were different in four major histologic subtypes, and the lowest levels were detected in CCC samples. Therefore, we selected ES-2 cell line, derived from a CCC, for *TET1*-overexpression experiments, and demonstrated that this leads to an increase in 5hmC levels and the inhibition of ovarian cancer growth *in vivo* and *in vitro*. Additionally, this indicates that TET1 activity and 5hmC levels play important roles in ovarian cancer development and progression, and that 5hmC levels may represent a valuable biomarker for the diagnosis of ovarian cancer.

Zelic *et al.* [32] showed that the hypermethylation in the promoter regions of tumor suppressor genes is usually observed in cancer, but global DNA hypomethylation can be observed in cancer tissues as well. Promoter regions of some genes were shown to be methylated in epithelial ovarian cancers, such as Fanconi anemia complementation group F (*FANCF*) and *RASSF2A* genes [33, 34]. RASSF5/NORE1 is an important tumor suppressor, which can inhibit tumor growth by promoting G1/S arrest [35, 36]. Additionally, this molecule is a RAS-proapoptotic factor, which can induce apoptosis by activating p53 pathway [37]. However, RASSF5 is frequently silenced in human cancers through promoter methylation, and the induction of RASSF5 expression was shown to inhibit cancer cell growth in humans [38]. Here, we showed that the expression of *RASSF5* is significantly increased in *TET1*-overexpressing cells, in comparison with that in the controls, and that the methylation levels of *RASSF5* promoter region are increased in ovarian cancer cells, which agrees with the results obtained in the previous studies. TET1 overexpression was shown to increase *RASSF5* promoter demethylation, which further stimulates *RASSF5* expression, leading to the suppression of ovarian cancer cell proliferation and colony formation. This indicates that *RASSF5* gene may represent a direct TET1 target, although it is not completely clear whether TET1 directly interacts with *RASSF5*, or through the interaction with other bridge proteins. Further analyses confirmed that *RASSF5* expression increases in *TET1*-overexpressing ovarian cancer cells, but the obtained results suggested that other TET1 downstream targets may also mediate the effects of this molecule on ovarian cancer cell proliferation and colony forming ability. Another possibility is that 5hmC provides a platform for the recruitment of TET1 to specific chromatin regions. Further delineation of the mechanistic link between TET1, RASSF5, and 5hmC production should allow a better understanding of TET1 functions in ovarian cancer cells.

Our results for TET1 expression in ovarian cancer cell have clinical and therapeutic implications. 1) Several reports have found a correlation between reduce 5hmC and poor patient prognosis in various cancers, such as malignant glioma and colorectal cancer [39, 40]. We also have observed TET and 5hmC protein levels were dramatically decreased in ovarian surface epithelial cancer tissues. These results indicate that TET1 down-regulation might be a key step initiating ovarian cancer and could possibly be used as an early diagnostic marker for ovarian cancers. 2)In our study, we also confirmed that TET1 overexpression increase chemosensitizing effects of paclitaxel and cis-platinum, which are first-line anticancer agents for human ovarian cancer (date not shown). In addition, TET1 overexpression activated DNA damage responses and apoptosis pathway with paclitaxel treatment, as shown by accumulations of pH2AX (Figure 7E) and cleaved caspase-3 (data not shown). These results show that TET1 overexpression sensitize ovarain cancer cell to chemotherapeutic drug treatment. Thus, small molecules that can increase TET1 activity may have the therapeutic potential to enhance the effect of chemotherapy for ovarian cancer patients.

## MATERIALS AND METHODS

### Plasmids and cell lines

Expression constructs encoding for *TET1 or TET3* C-terminal cysteine-rich dioxygenase (CD) domain (Flag-TET1 or Flag-TET3) and activity-dead mutant *TET1* (TET1-MU) or *TET3* (TET3-MU) were kindly provided by Dr. Heng-yu Fan.

Human ovarian cancer cell lines, SKOV3, A2780, ES-2, HO8910, OV2008, C13, 293T, and Hep3132 were purchased from ATCC. The immortalized mouse ovarian surface epithelium (mOSE) were reported previously [41]. All cell lines were cultured under an atmosphere of 5% CO_2_ at 37°C. All cells were cultured in Dulbecco’s Modified Eagle Media( DMED, Gibco) supplemented with 10% fetal bovine serum (FBS; Gibco) and 1% penicillin-streptomycin solution (Gibco). The chemotherapeutic drugs used in this study included paclitaxel (0.01 μg/mL) (Bristol-Myers Squibb, New York, NY, USA). MLN4924 (MedChemExpress) was applied in the concentration of 1 μM. All reagents were used according to the instructions provided by the suppliers.

### TET1-overexpressing ovarian cancer cell lines

ES-2 and A2780 were infected by lentiviruses carrying empty vectors (LV-CON) or green fluorescent protein (GFP)-TET1-Flag (LV-TET1). Briefly, 293T Phoenix retrovirus packaging cells were transfected with empty vectors and GFP-TET1-Flag expressing plasmids. Forty-eight hours after the transfection, retroviral supernatant was supplemented with 5 μg/mL polybrene, filtered through a 0.45-μm filter, and used for infection. Ovarian cancer cells were seeded in 60-mm dishes and infected with lentivirus carrying *TET1* or control vector, and 48 h later, they were selected with 5 μg/mL puromycin in the culture medium.

### Colony formation assay

Ovarian cancer cells (500 cells/dish) were seeded in 60-mm dishes for approximately 10 days and the culture medium was replaced with fresh medium every 3 days. All experiments were performed in triplicates, and plates were incubated in a 5% CO_2_ humidified atmosphere at 37°C. Colonies were stained using Coomassie Brilliant Blue and counted 10 days after plating.

### RNA interference

Ovarian cancer cells were transfected with different siRNAs using Lipofectamine RNAiMAX Reagent (ThermoFisher Scientific). Briefly, cells were seeded in six-well plates and transfected with siRNA and Lipofectamine RNAiMAX Reagent, each incubated separately in Opti-MEM for 5 min, mixed together for 10 min at room temperature, and then the mixture was applied to the cells plated in 1 mL of medium (final siRNA concentration, 80 nM). The siRNA sequences used in this experiment (GenePharma, Shanghai, China) were as follows:

siCON(siNC): UUCUCCGAACGUGUCACGUTT.

si*TET1*: CAGGUGGGUUUGCAGAAACAA.

si*ROC1*: GACUUUCCCUGCUGUUACCUAATT.

si*DCAF1*: UCACAGAGUAUCUUAGAGATT.

si*DDB1*: GGCCAAGAACAUCAGUGUGTT.

### Cell growth assay

Cells were seeded in 96-well plates at a density of 5×10^3^ cells/well in DMEM containing 10% FBS. After they had adhered, cell growth was monitored for 48 or 72 h, and then assessed using MTT assay. Briefly, 20 μL of MTT solution (5 mg/mL in PBS) was added into triplicate wells and cells were incubated for 4-6 h in an incubator. Absorbance at 490 nm was determined using a microplate reader.

### Mice and xenograft models

Mice (n=15) were housed under standard conditions, under a 14 h/10 h light/dark schedule, and they were provided food and water *ad libitum.* All animal protocols were in accordance with the NIH Guide for the Care and Use of Laboratory Animals. To assess cancer cell proliferation *in vivo*, ovarian cancer cells (1×10^6^) were subcutaneously transplanted into both back flanks of 8-week-old female nude mice. Four weeks later, primary tumor masses were excised and fixed overnight in 10% PBS-buffered formalin, dehydrated using an ascending series of graded ethanol solutions, and then embedded in paraffin.

### Immunohistochemical (IHC) analysis

Paraffin-embedded human tissues from ovarian tumors were provided by the Jiaxing Maternity and Child Health Care Hospital, China. Paraffin-embedded human ovarian cancer tissue microarrays (TMAs) were purchased from Fanpu Biochec, Inc. Each patient specimen in these TMAs was represented in two cores on the slide, each measuring 1.5 mm in diameter. Clinical information was provided by the commercial source. The use of archived cancer samples for this study was approved by the Jiaxing University Institutional Review Board.

Primary tumor masses were cut into 5- μm tick sections with a Leica RM2235 microtome and stained with hematoxylin and eosin (H&E). For immunochemistry, sections were deparaffinized and rehydrated with xylene and an alcohol gradient. Afterwards, the sections were incubated in 0.3% H_2_O_2_. After the antigens were retrieved using 10 mM sodium citrate (pH 6.0), the sections were incubated with anti-5hmC (Active Motif), anti-ROC1, anti-DDB1 (Epitomic), anti-DCAF1 (ProteinTech), anti-pH2AX, anti-cleaved caspase 3 (Cell Signaling Technology), and anti-phospho-histone H3 (Epitomics) antibodies (1:200) using a Vector ABC kit (Vector Laboratories), at room temperature for 1 h, which was followed by a reaction with biotin-labeled goat secondary antibodies for 30 min. Staining was performed using Vectastain ABC kits and 3,3′-diaminobenzidine (DAB) peroxidase substrate kits (Vector Laboratories, Burlingame, CA, USA). Staining intensity of 5hmC, ROC1, DDB1, DCAF1, pH2AX, cleaved caspase 3, and phospho-histone H3 was scored by a pathologist (L. Guo).

### TUNEL assay

Terminal deoxynucleotidyl transferase-mediated dUTP nick-end labeling (TUNEL) assays were performed with 10% formalin-fixed paraffin-embedded sections, using an Apop Tag Plus Peroxidase *In Situ* Apoptosis Detection Kit (Serologicals Corporation, Norcross, GA, USA), according to the manufacturer’s instructions.

### Immunofluorescence analysis

Cultured cells were seeded on cover slips and transfected with siCON, si*TET1*, or GFP-TET1-Flag plasmids. After 24 h, cells were washed with PBS, and fixed for 10 min at room temperature with 4% paraformaldehyde in PBS.

Primary tumor masses were fixed in 4% paraformaldehyde, embedded in O.C.T. compound (Sakura Finetek USA, Inc.), and stored at -80°C before preparing 7-μm sections using a Leica CM1950 cryomicrotome (Leica Microsystems, Wetzlar, Germany).

Cells and sections were permeabilized with 0.3% Triton X-100 in PBS, incubated with the blocking buffer (PBST containing 5% bovine serum albumin (BSA)), and were sequentially probed with anti-5hmC (Active Motif), anti-pH2AX, anti-cleaved caspase 3 (Cell Signaling Technology), and anti-phospho-histone H3 (Epitomics) antibodies (1:200), which was followed by a reaction with Alexa Fluor 594- or 488-conjugated secondary antibodies (Molecular Probes). Slides were mounted using VectaShield and stained with 4′, 6-diamidino-2-phenylindole (DAPI, Vector Laboratories). Digital images were acquired using an epifluorescence microscope (Nikon Eclipse 80i) at 4-100× magnification.

For 5hmC staining, cultured cells were fixed in PBS-buffered 4% paraformaldehyde for 30 min, and then treated with 4 N HCl for 15 min at room temperature, in order to denature double-stranded DNA. The samples were extensively washed with PBS and then blocked with 5% BSA in PBS for 1 h. Subsequently, samples were incubated with anti-5hmC primary antibody (1:400) at room temperature for 1 h. Alexa Fluor 594- or 488-conjugated secondary antibodies were used to detect the signals. The samples were mounted and images were acquired as described above.

### Western blot analysis

Total proteins were isolated from the cell extracts, and 30 μg of protein were separated by SDS-PAGE and transferred to polyvinylidene difluoride (PVDF) membranes (Millipore, Bedford, MA, USA). After probing with primary antibodies, membranes were washed in Tris-buffered saline that contained 0.05% Tween-20 (TBST) and incubated with a horse-radish peroxidase-linked secondary antibody. Finally, the obtained bands were detected using an Enhanced Chemiluminescence Detection Kit (Amersham). The primary antibodies used were as follows: anti-FLAG (Sigma-Aldrich), anti-p-H2AX (Ser-139) (Cell Signaling Technology), anti-ROC1 (Epitomics), anti-DDB1 (Epitomics), anti-DCAF1 (ProteinTech), anti-RASSF5 (Abcam), anti-TET1 (Abcam) and anti-ERK1/2 (Santa Cruz Biotechnology) antibodies (1:1000).

### RNA extraction and real-time RT-PCR analysis

Total RNA was extracted using Trizol (Invitrogen), according to the manufacturer’s instructions. Real-time PCR analysis was performed using Q Tag SYBR Green Master Mix (Becton Dickinson Medical Devices, USA) and an Applied Biosystems 7500 Real-Time PCR System. Relative mRNA levels were determined by normalizing the obtained expression levels to the endogenous β-actin mRNA levels, using Microsoft EXCEL. For each of the indicated genes, the relative transcript levels of the control sample were set at 1, and the transcript levels of other samples were compared with that of the control. Quantitative RT-PCR reactions were performed in triplicates. The following primers were used to amplify target genes:

*β-actin*: 5′-GCTCTTTTCCAGCCTTCCTT-3′; 5′-GTACTTGCGCTCAGGAGGAG-3′.
*TET1*: 5′-AAGTGAAACCGAACCCCATT-3′; 5′-ACTGAGTCTTCCCGAAGGCAT-3′.
*CYP24A1*: 5′-CAGAGCTTCAACTGCATTTGG-3′; 5′-TGTAGAATGCCTTGGATCCC-3′.
*FOSB*: 5′-GAAAGCCTTTCCTCGGTCTC-3′; 5′-TTCCTGGAGAGGATGTGAGG-3′.
*PCDH10*: 5′-AGAATTACTGCTATCAGGTATGCCT-3′; 5′-TCGTTGGACAAAATGCTTCC-3′.
*RASSF5*: 5′-CACTGCGACTCTGGGATCTC-3′; 5′-TCACTTCCTGGCAGGAGAAG-3′.
*THBS2*: 5′-TTCTGTTTCTGTGTTGTGGGG-3′; 5′-AGTGCAGGGTTTCAGTGGTG-3′.

### Bisulfite sequencing

DNA methylation profile was analyzed using bisulfite sequencing, with EpiTect Plus DNA Bisulfite Kit, according to the manufacturer’s instructions. Briefly, genomic DNA was extracted from cells and bisulfite DNA conversion was performed using a thermal cycler. Following this, the bisulfite converted DNA was purified and used for PCR analyses. Specific PCR products were recovered using a Gel Extraction Kit (Axygen) and cloned into pUCm-T vectors (Sangon) for sequencing. The primers in Table 2 were used to amplify target genes.

**Table 2:** Primers used to amplify gene targets

Gene name	Sequence (5′-3′)
*CYP24A1*	Forward (F)1: AGTTTAGGTTGGGGGTATTTGG
	Reverse (R)1: ACAAAACATACCCTAAATAACCAATAAAC
	F2: AATATGGAGAGGGATAGATAGGAGGA
	R2: ACTCTTACTAATAAAAAAACTCATAACAA
*FOSB*	F1: TTGATGTTATTGTTAGGATATTAAATAAATATTT
	R1: AAATCTTTCCAAAAACTTTCTCCC
	F2: GGGAGAAAGTTTTTGGAAAGATTT
	R2: CCTAAACACAAAAAAACCCCTATAA
*PCDH10*	F1: GAGGTGTTGGATATTAATGATAATTTTT
	R1: AACACATTATCATTAAAATCCAACACT
	F2: AGTGTTGGATTTTAATGATAATGTGTT
	R2: AAACCACCACAATACCGAACG
	F3: CGTTCGGTATTGTGGTGGTTT
	R3: TAAAAACTAAAATCCTTCAACTACTCATAAT
	F4: ATTATGAGTAGTTGAAGGATTTTAGTTTTTA
	R4: CCACCAACTAAACCACCAAAATAA
*RASSF5*	F1: TGAAGGAAGGGGAAATTTAATTAGAG
	R1: TCTTCACCTAAAACAACTACAAAATTC
	F2: GAATTTTGTAGTTGTTTTAGGTGAAGA
	R2: CAATCTCTACTACAAACCAAAC
	F3: GTTTGGTTTGTAGTAGAGATTG
	R3: TACCCCAAAACCCTCCCAATCCTT
	F4: TGATTTGTAGTTTTTTGAGTTTATTTGTTAT
	R4: AAAAAAAATAAACACCCCTCCC
	F5: GGGAGGGGTGTTTATTTTTTTT
	R5: TACAATACCCACTACTCATACTACTATCCA

### Dot-blot assays

In order to detect 5hmC levels in genomic DNA, we extracted DNA, and sample concentrations were adjusted. DNA samples were denatured at 99°C for 10 min, and immediately cooled on ice. The resulting DNA was bound to a nitrocellulose membrane, air dried, and cross-linked using the UV light. Immunoblotting was performed using the anti-5hmC antibodies followed by the incubation with horseradish-peroxidase-linked secondary antibodies.

### Statistical analysis

All results are reported as mean ± standard error of mean (SEM). Each experiment was performed in triplicate and was repeated at least three times. The results obtained in two experimental groups were compared by two-tailed unpaired Student's t tests. p<0.05, p<0.01, and p<0.001 were considered statistically significant, and are indicated in the figures by one, two, and three asterisks, respectively.

## SUPPLEMENTARY MATERIALS FIGURE AND TABLES






