# Supplementary material for: TET1 inhibits cell proliferation by inducing RASSF5 expression
Source: Oncotarget. 2017 Sep 23;8(49):86395–409. doi: 10.18632/oncotarget.21189 (PMC5689693; doi:10.18632/oncotarget.21189)
Supplement: Supplementary file 1 [file oncotarget-08-86395-s001.pdf]

TET1 inhibits cell proliferation by inducing RASSF5 expression

SUPPLEMENTARY MATERIALS

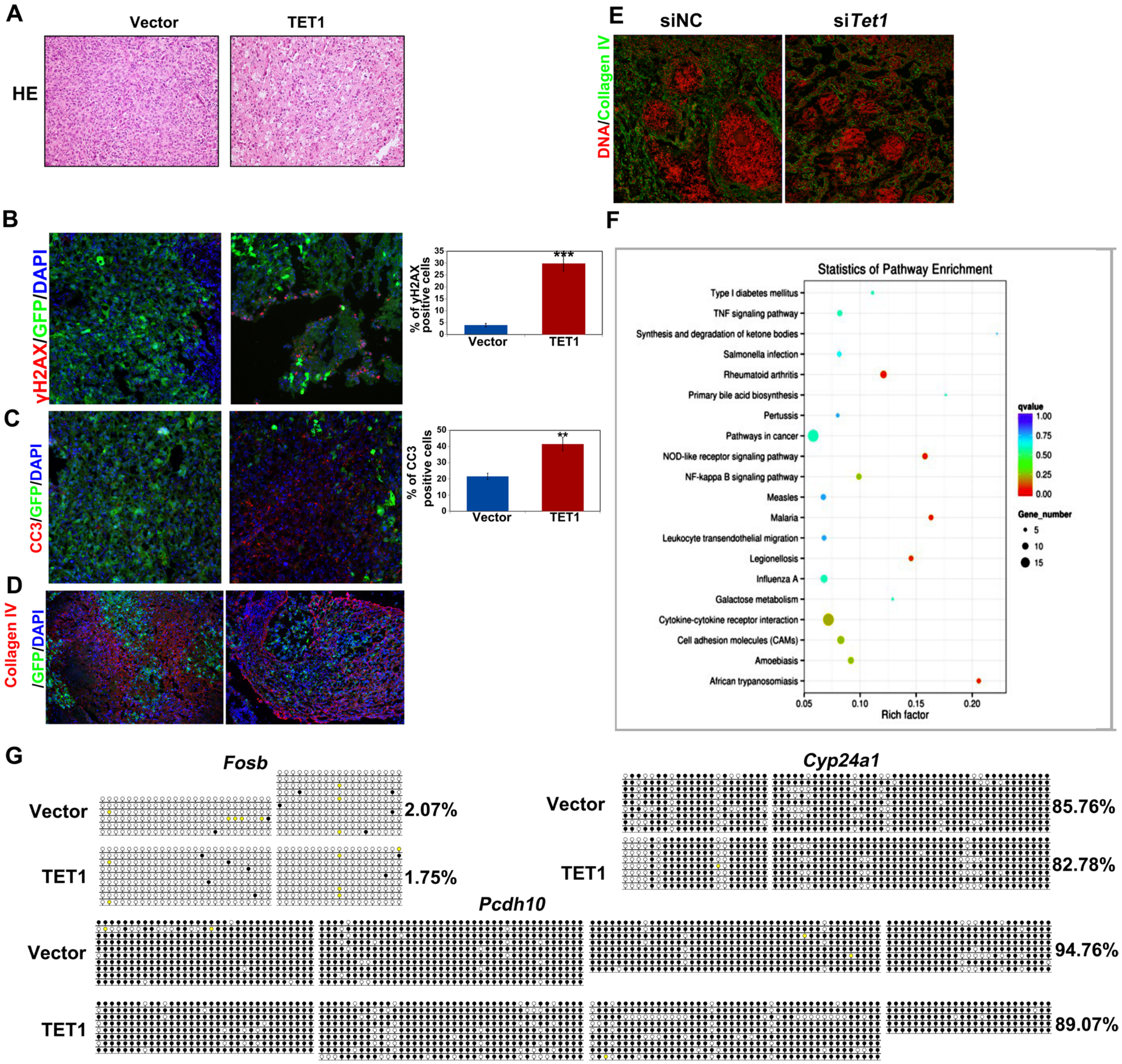

Supplementary Figure 1: DNA promoter methylation in *TET1*-overexpressing ovarian cancer cells. **(A)** Control or *TET1*-overexpressing ES-2 cells were injected into mice, and the resulting tumors were paraffin-embedded and stained with H&E. **(B-E)** Immunofluorescence analysis of γH2AX, cleaved caspase 3, and collagen IV expression (**red**). **Green**, TET1 overexpression. **Blue**, DAPI-stained DNA. **(F)** Genes upregulated following *TET1*-overexpression are involved in different signaling pathways. **(G)** *CYP24A1*, *FOSB*, and *PCDH10* promoter methylation in *TET1*-overexpressing ES-2 cells. Open and filled circles represent unmethylated and methylated CpG islands, respectively. The percentages of methylated CpGs are indicated.

Supplementary Table 1: Human ovarian cancer tissue microarray I

See Supplementary File 1

Supplementary Table 2: Human ovarian cancer tissue microarray II

See Supplementary File 2
